# Rotavirus A in wild and domestic animals from areas with environmental degradation in the Brazilian Amazon

**DOI:** 10.1371/journal.pone.0209005

**Published:** 2018-12-18

**Authors:** Bruno de Cássio Veloso de Barros, Elaine Nunes Chagas, Luna Wanessa Bezerra, Laila Graziela Ribeiro, Jose Wandilson Barboza Duarte Júnior, Diego Pereira, Edvaldo Tavares da Penha Junior, Julia Rezende Silva, Delana Andreza Melo Bezerra, Renato Silva Bandeira, Helder Henrique Costa Pinheiro, Sylvia de Fátima dos Santos Guerra, Ricardo José de Paula Souza e Guimarães, Joana D'Arc Pereira Mascarenhas

**Affiliations:** 1 Evandro Chagas Institute, Ministry of Health, Ananindeua, Pará, Brazil; 2 Amazon Metropolitan University Center, Belém, Pará, Brazil; 3 University of the State of Pará, Belém, Pará, Brazil; 4 Federal University of Pará, Belém, Pará, Brazil; University of Hong Kong, HONG KONG

## Abstract

Acute gastroenteritis is one of the main causes of mortality in humans and young animals. Domestic and mainly wild animals such as bats, small rodents and birds are highly diversified animals in relation to their habitats and ecological niches and are widely distributed geographically in environments of forest fragmentation in some areas of the Amazon, being considered important sources for viruses that affect humans and other animals. Due to the anthropical activities, these animals changed their natural habitat and adapted to urbanized environments, thus representing risks to human and animal health. Although the knowledge of the global diversity of enteric viruses is scarce, there are reports demonstrating the detection of rotavirus in domestic animals and animals of productive systems, such as bovines and pigs. The present study investigated the prevalence of Rotavirus A in 648 fecal samples of different animal species from the northeastern mesoregion of the state of Pará, Brazil, which is characterized as an urbanized area with forest fragments. The fecal specimens were collected from October 2014 to April 2016 and subjected to a Qualitative Real-Time Polymerase Chain Reaction (RT-qPCR), using the NSP3 gene as a target. It was observed that 27.5% (178/648) of the samples presented positive results for RVA, with 178 samples distributed in birds (23.6%), canines (21.35%), chiropterans (17.98%), bovines (14.6%), horses (8.43%), small rodents (6.74%), pigs (3.93%) and felines (3.37%), demonstrating the circulation of RVA in domestic animals and suggesting that such proximity could cause transmissions between different species and the occurrence of rearrangements in the genome of RVA as already described in the literature, associated to the traces of environmental degradation in the studied areas.

## Introduction

Emerging and reemerging infectious diseases are increasing each year in several countries, with an impact both on human populations and on domestic and wild animals living in areas with considerable forest remnants [1]. Most of these diseases are of viral origin, suggesting the emergence and reemergence of viruses that are triggered by human activities that modify the environment [2].

The populations of wild animals that inhabit forest fragments are strategic groups for studies of public health and the transmission of zoonosis, given that they act as indicators in the assistance and intervention in the human populations, aiming at the prevention of outbreaks and epidemics [3].

Acute gastroenteritis can be caused by infection in the gastrointestinal tract, caused by different infectious or parasite agents [4–7]. They represent one of the main causes of mortality in humans, and in young animals, counting for about 25% of mortality [8]. Rotavirus is widely distributed in animals, which act as sources of rotavirus emergent strains, with these animals acting in the transmission between species and through reassortment leading to the emergence of new strains which have been reported in human infections [9–12].

The rotavirus (RV) belongs to the *Reoviridae* family and comprises nine species known as Rotavirus group A to I, with a recent proposal of the J species [13, 14]. Rotavirus A (RVA) is widespread worldwide and predominantly infects humans, bovines and other mammal species, as well as birds [15]. They have a double-stranded ribonucleic acid (dsRNA) genome, divided into 11 segments coding for structural proteins (VP1-VP4, VP6 and VP7) and non-structural (NSP1-NSP5/NSP6) proteins [16, 17].

There are records of a close relationship between Amazonian wildlife and human populations [18], and this interaction is the effect of anthropogenic urbanization activities that result in the deforestation of forest areas, causing the degradation of previously isolated sites such as caves and small caves, a continuous and nature progressive process that has led not only to changes in wildlife habitats but also to a greater relationship with human populations in rural and urban environments, contributing to the occurrence and emergence of diseases different from what normally occurs in endemic regions [19–22].

Although the results of RVA have already been described globally [12, 23–30], in Brazil, the occurrence, diversity and role of rotavirus in these animals are still poorly studied, considering the large number of present species [4, 31–34].

In the Brazilian Amazon, especially in the state of Pará, the city of Belém and Northeast metropolitan mesoregions are some of the areas with the highest indexes of environmental changes [35], which are concentrated, along with the fact that the knowledge of the global diversity of enteric virus in animals is scarce [36].

Therefore, it is important to monitor the health of domestic and wild animals in their natural habitat, especially in areas with anthropic alterations that have an interface with rural communities and enterprises, in order to investigate the occurrence of RVA in this population. These communities are ecologically complex, because they have multiple hosts and endless pathogens that may eventually circulate in contiguous urban centers, in addition to the fact that it should also be considered that there is still a lack of studies showing the significance of these viruses infecting this population, as in the context of epidemiological surveillance, these animals become important, since they can be considered as natural sources, with the possibility of transmission to humans [37–39].

The qualitative real-time polymerase chain reaction (qRT-PCR) used the NSP3 gene and the TaqMan probe from a highly conserved region of the rotavirus non-structural protein 3 (NSP3), which was previously used in samples from human origin and with low viral loads according to studies conducted by Zeng et al. [40] and Mijatovic et al. [41]. In the present study, this assay was adopted as a screening test for the detection of RV in asymptomatic animal populations.

Therefore, the objective of the present study was to estimate the number of animals infected by RVA in the forest environment and the possible environmental impacts, and the geographical distribution of this agent in the region.

## Material and methods

### Study area

**Fig 1:**
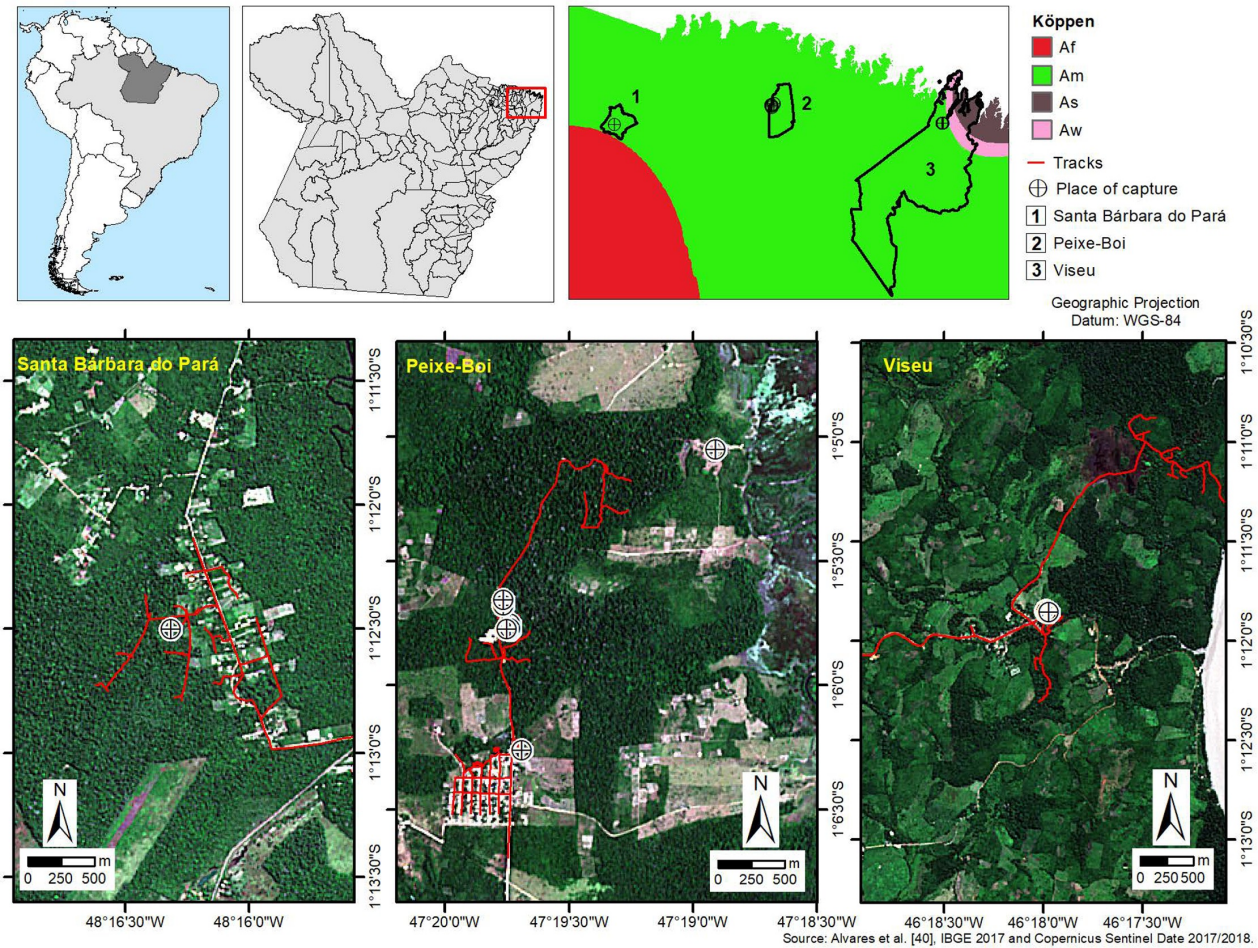
Place of study and capture of the animals.

The study area included the settlements of Expedito Ribeiro (Santa Bárbara do Pará), Vila Ananin (Peixe-Boi) and Açaiteua-Centro Alegre (Viseu), located in areas with environmental degradation in the state of Pará, in the Brazilian Amazon. According to Alvares et al. [42], the city of Santa Bárbara do Pará presents a tropical type of motion-type Am and Viseu presents atype of motion, with dry seasons in summer and winter-Am, As and Aw (Fig 1), taking into account the weather forecast classification of Köppen-Geiger.

The collection points of the positive samples were georeferenced and the data were transported to the ArcGIS 10.3 program for the construction of distribution maps for the RVA at the interface between agroecosystems and natural ecosystems.

### Data extraction and geoprocessing analysis

Precipitation data were obtained from The Brazilian National Institute of Meteorology (Inmet - http://www.inmet.gov.br/) for the years of capture in the Expedito Ribeiro Settlement (2014) and Açailândia (2015) of the Data Collection Platforms (PCDs) of Belém, located 50 km from Santa Bárbara do Pará, and Tracuateua, located 50 km from Peixe-Boi and 100 km from Viseu.

Garmin GPSMap 64s Global Positioning System (GPS) coordinates were collected in the field.

The municipal boundaries were obtained on the website of the Brazilian Institute of Geography and Statistics (IBGE) (http://www.ibge.gov.br/) and data on deforestation and land use were obtained from the PRODES [43] and TerraClass [44] Projects. PRODES has annual data in digital format since 2000 and TerraClass presents biannual data since 2004.

The satellite image was generated using the sensor Sentinel 2 of the European Space Agency (ESA) (https://sentinel.esa.int/ web/sentinel/user-guides/sentinel-2-msi) with Open Access CC-BY License (http://open.esa.int/) from the years of 2017 and 2018.

All the data obtained was stored in a Geographic Database (BDG). The BDG was imported/stored in a GIS for the editing of the graphic elements, establishment of topological relations between the elements and their respective attributes, spatial analysis and visualization of the result through thematic maps.

### Choice of study areas

For the present study, forest fragments of similar size, shape and Phyto physiology were chosen, considering an open peri urban matrix with similar soil use. The selected fragments were distributed within the mesoregions studied, and in each selected fragment fecal samples were randomly collected from domestic and wild animals [45].

Soil use classes were obtained from the TerraClass data mosaic from 2004 to 2016, because the study sites were in an area with a high cloud presence, which prevented observation (the area was not observed).

The data processing, interpretation, visualization and spatial analysis were performed in ArcGIS software (http://www.arcgis.com/).

For the analysis of data related to the determination of the richness, composition and abundance of the fauna of the animals studied in the study area, considering the collection methods adopted and the species available in each city, each sample was considered as an independent sample.

### Statistical analysis

The richness of wild fauna and domestic animals was determined by the total number of species including all collection methods, and the similarity of species was made by the chi-square analysis between the samples of the different treatments with the aid of the EstimateS 8.0 software [46].

For the calculation of the Test T, the Statistica software was used, and the indices of infected animals in the two environments (forest fragment and peridomicile) were calculated for each treatment sampled by collection area, using the software Past 1.92. Aiming at comparing the values of the diversity indexes through the paired test, as well as the descriptive analysis of the anthropic effects [47].

The data obtained for the occurrence of RVA and the questionnaires was inserted into a database for a descriptive analysis of the epidemiological profile of the animal population in the three forest ecosystems studied. In this analysis, descriptive statistical treatments were carried out, using customized "row-columns" type charts, referring to the data, in order to characterize the sample and quantify the results using absolute frequency values using the chi-square test and the Test T.

### Ethical aspects

#### Population study, collection of clinical specimens and laboratory methodology

The flying animals (wild birds and chiroptera) were captured using mist nets which were opened at dawn (4:00 a.m.) and closed in the morning (9:00 a.m.) and were inspected every one hour until the closing, with a sampling effort of 15 days. This research was approved by National Council for Animal Control and Experimentation (CONCEA), System of Authorization and Information in Biodiversity—SISBIO/ICMBIO/Ministry of the Environment under No. 37174–1 and by Committee on Ethics in Animal Use (CEUA / IEC) No. 019/2014.

All procedures with animals were performed by veterinarians, being birds and bats identified and released at the same capture site. The fecal specimens were collected by stimulation of the rectal ampulla with the use of a "Zaragatoa", packed in cryogenic vials, identified, stored in liquid nitrogen, and later sent to the Laboratory.

Wild animals (small non-flying mammals) were trapped within live-containment traps of the Tomahawk cage (size 45x16x16cm) and Sherman type aluminum (size 30x9x8cm). In each sample plot, 61 traps were distributed, 20 Shermans and 41 Tomahawks being baited with a mixture made with peanut paste, sardines, cod liver oil and corn meal, as well as fruit like banana, apple and pineapple. All the traps used were inspected daily in the morning, the baits being exchanged when necessary and later after the capture in bags of cloth and at least five specimens of each species were chosen for the collection of biological material. The wild animals were sedated with a combination of ketamine 20mg/kg and xylazine 2mg/kg intramuscularly and subsequently, euthanized with anesthetic overdose of 2% lidocaine in the foramen magnum, according to the recommendation of the National Council for the Control of Animal Experimentation (CONCEA).

From October 2014 to April 2016, 1,282 fecal samples were collected from wild and domestic animals. Amongthese, 648 (50.5%) samples were randomly selected for RVA research and handled in Level Three Biosafety Laboratory (NB3).

The viral genome was extracted using the TRIZOL LS REAGENT protocol (INVITROGEN, USA/KIT QIAGEN), following the manufacturer's recommendations, with minor adaptation according to the protocol described in the supplemental data.

The qRT-PCR was conducted according to Zeng et al. [40] for the detection of RVA using the NSP3 segment of RVA as the target gene sequence. The assay was conducted in a mixture containing: RNAse-free H_2_O, TaqMan RT-PCR Mix (2x), TAqMan RT Enzyme Mix (40x), primers for the NSP3 gene, Primer NSP3 Forward (20mM), Primer NSP3 Reverse (20mM), probe NSP3 S (10nm), Template (RNA) 3μL, having a total reaction volume of 17μL and reverse transcription cycling of 50°C, 30 minutes, denaturation of 95°C, 10 minutes, annealing of 45 cycles of 95°C, 15 seconds and extension of 60°C, 1 minute.

The analyzes were considered positive when presenting the cycle threshold (CT) ≤ 40. In order to guarantee a reliable test result, the measurements of contamination control were performed with the use of positive animal control (SA11 prototype) and a negative control (ultrapure water).

All RVA-positive samples were subjected to reverse transcription-polymerase chain reaction (RT-PCR) according to Mijatovic et al [41] to genotyping low viral loads samples. First round was performed with consensus primers N-VP4F1/N-VP4R1 and the Nested-PCR was conducted with N-VP4F2/N-VP4R2 primers to amplify VP4 gene. Amplicons were purified and sequencing for VP4 gene using the same primers of Nested-PCR. The sequences were collected from an automated ABI Prism 3130xl DNA sequencer (Applied Biosystems). The sequence fragments were assembled and edited using the Geneious Bioinformatics software platform v.8.1.7. Posteriorly, the data were compared with othersequences from the National Center for Biotechnology Information GenBank database using BLAST alignment tool to elucidate the RVA genotype of the samples.

## Results

From October 2014 to April 2016, a total of 648 fecal samples of wild and domestic animals belonging to three forest fragments areas were tested for the NSP3 gene by qualitative qPCR, and 178 (27.5%) were positive for RVA, distributed among the species: birds (23.6%), canines (21.35%), bats (17.98%), cattle (14.6%), horses (8.43%), small rodents (6.74%), swine (3.93%) and felines (3.37%). The CT interval ranged from 28.47 to 39.9 (mean of 36.79) with the following mean values per species: horses (34.11), birds (34.24), cattle (36.02), canines and chiropterans (37.1), rodents (37.76), swine (37.91) and felines (38.36).

It was possible to detect viral strains in all genders of animals studied and in the harvesting period none of the animals showed signs of acute infection and / or diarrhea.

**Fig 2:**
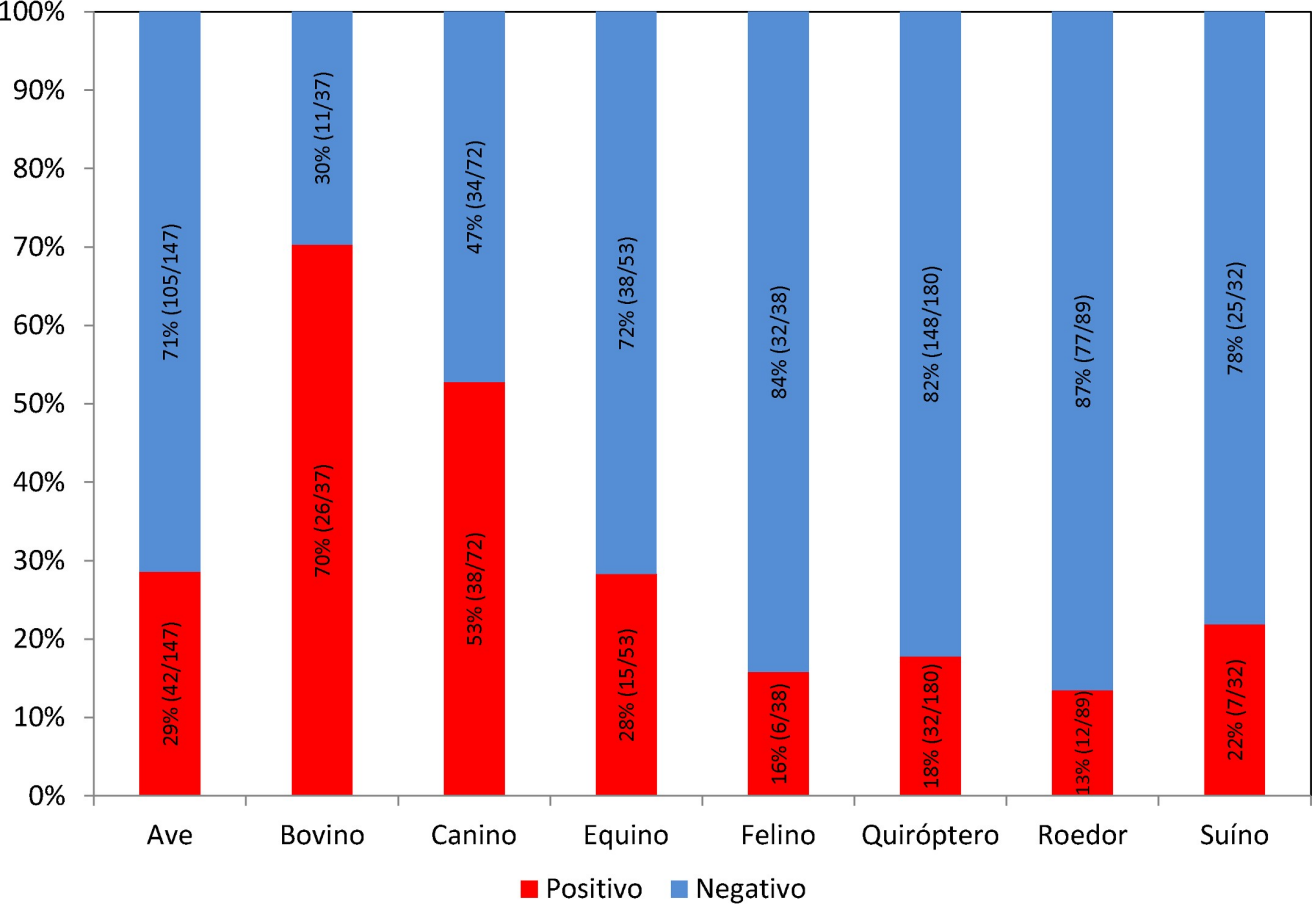
Frequency of Rotavirus A in domestic and wild animals in the Brazilian Amazon from 2014 to 2016.

Rotavirus A (RVA) detected in the present study of wild and domestic animals belonging to the three areas of forest fragment, according to Fig 2.

In relation to the evaluated bovines, only in the city of Viseu, these species were studied because they were created extensively. In addition, most of the animals were young with ages varying from 1 day to 8 yearsold, history of deficient vaccination, lack of technical assistance and raised in the form of subsistence. The animals showed no symptoms of diarrhea, only low weight performance and poor sanitary management status. In relation to chiroptera, 32 (17.98%) positive samples for RVA were distributed among *Carollia perspicillata* species, with 12 (37.5%) being all adults, 9 (28.12%) *Desmodus rotundus* samples (4 young and 5 adults), 5 (15.6%) of *Uroderma bilobata* (15.62%), 3 (9.37%) of *Artibeus lituratus* and the species *Artibeus Planirostus*, *Diaemus iyoug* and *Glossophagine* with 1 (3.12%) each. These animals came from areas of forest fragments located near bovine and equine farms, in addition to inhabiting small chicken farms.

**Fig 3:**
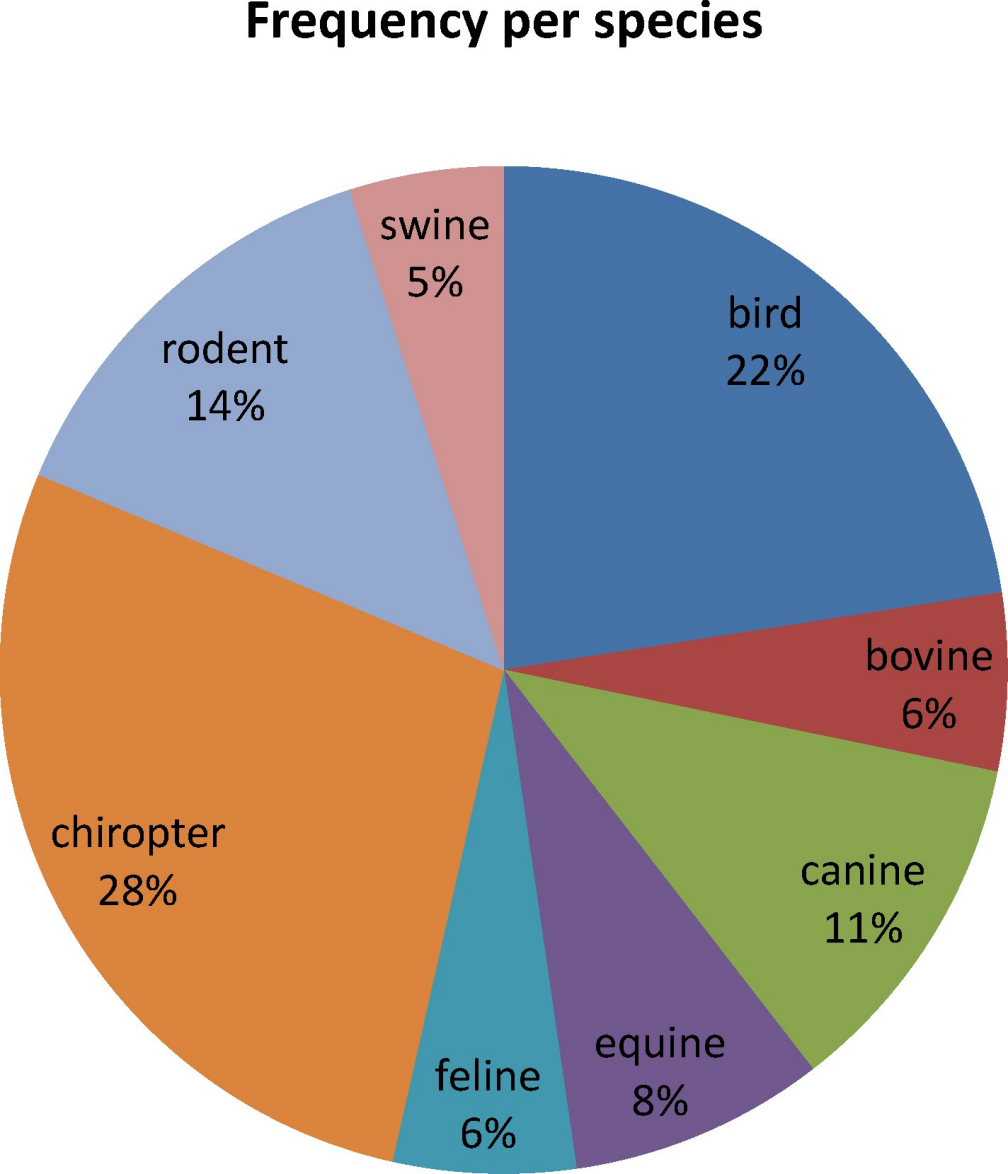
Frequency of Rotavirus A in domestic and wild animals in the Brazilian Amazon from 2014 to 2016.

Fig 3 shows the results obtained for all the species of animals investigated in the forest fragment as well as in the peridomicillus area.

**Fig 4:**
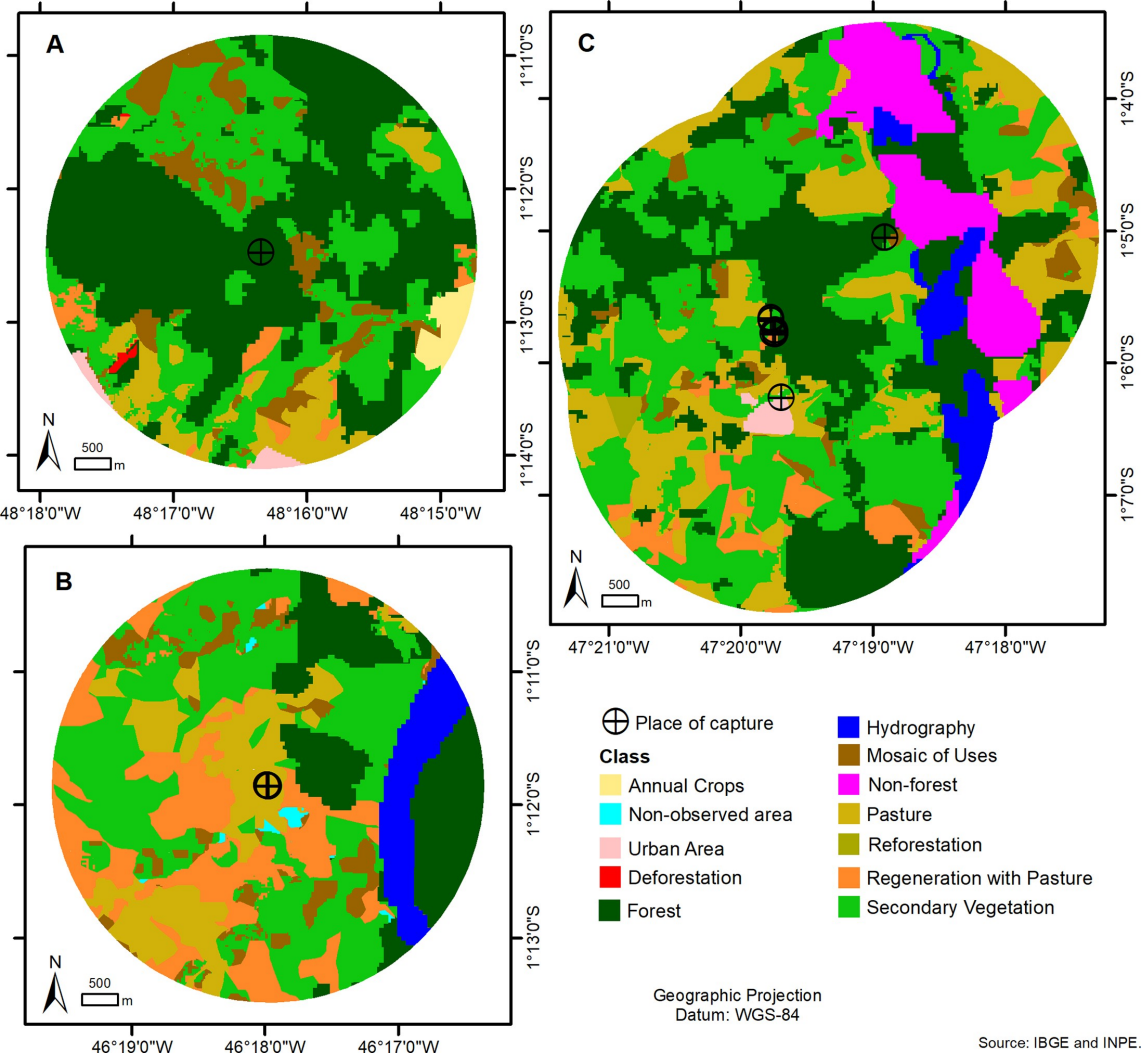
Map of distance of 3 km at the capture sites of the bats in (A) Santa Bárbara, (B) Viseu and (C) Peixe-Boi, with land use classes.

**Fig 5:**
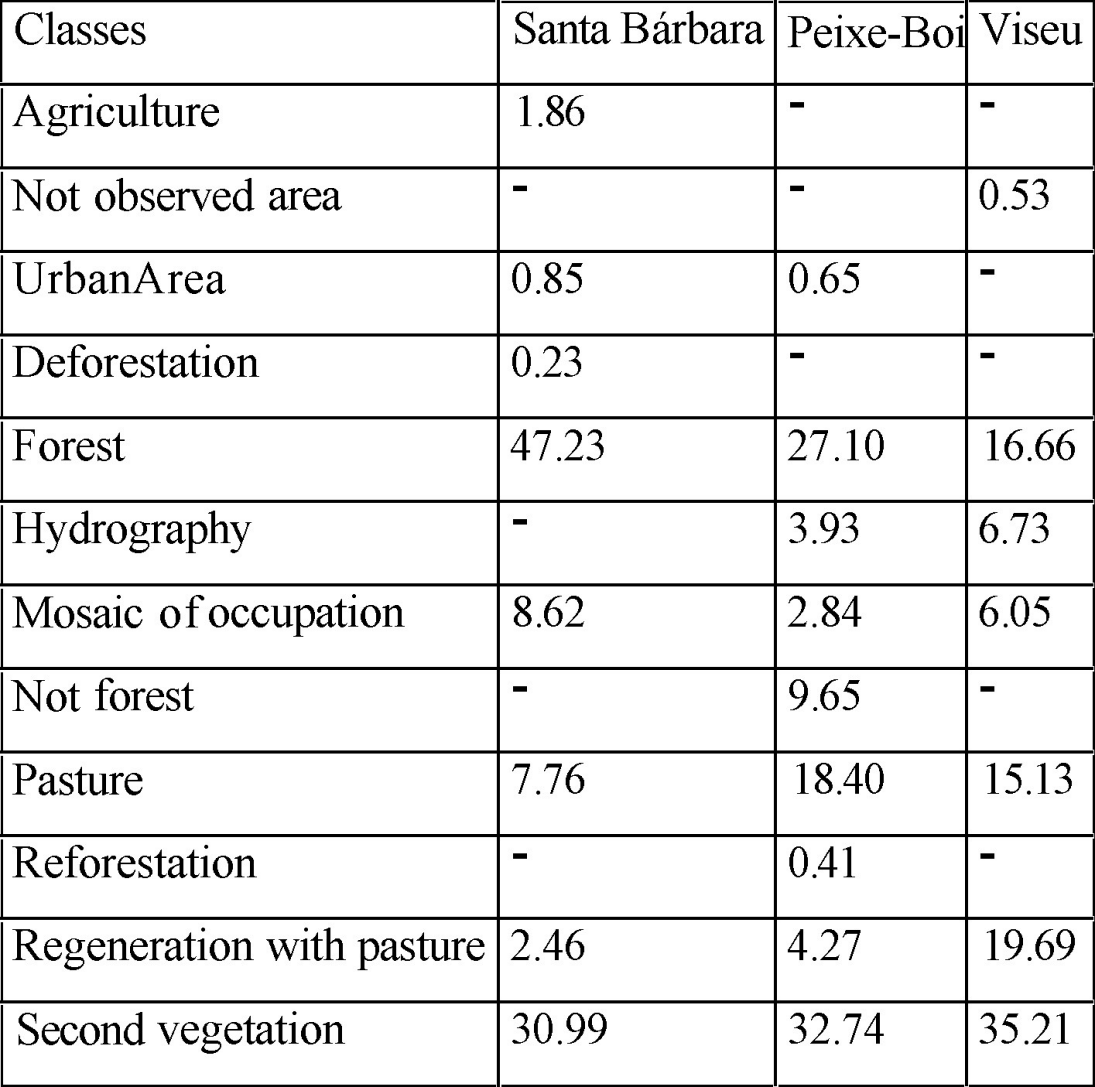
Shows the percentage of each class of soil use within a 3 km radius of the place of capture of the animals in each city studied.

The anthropic variables were analyzed for the three cities studied, as well as the use of the soil within the range of the animals, obeying the domicile, the peridomicile and the forest fragment where the traps of small rodents, birds and various species of animals were captured (Fig 4 and Fig 5).

Considering the factors related to the anthropic activities in the three studied areas within the three cities of the present study, it was observed that the city of Santa Bárbara is the one that has a better area of preserved forest and the city of Viseu a smaller area. However, in the city of Santa Bárbara, a greater concentration of occupations was observed around the area of forest fragment. It was observed in this chosen area of the city, the presence of different families living in a rural settlement, surviving from the exploitation of forest resources and the creation of small animals for subsistence, such as poultry and fish farming, as well as family farming products.

The breeding of animals in native pastures was only observed in the cities of Peixe Boi and Viseu. Extensive livestock farming was practiced with beef cattle, equines for work and small animals (swine and goats). In relation to the most preserved pasture area, the city of Peixe Boi had the largest area, according to the data shown in Fig 5, however, in the city of Viseu, a higher regeneration was observed in the pastures during the period of the study, with significant secondary vegetation.

**Fig 6:**
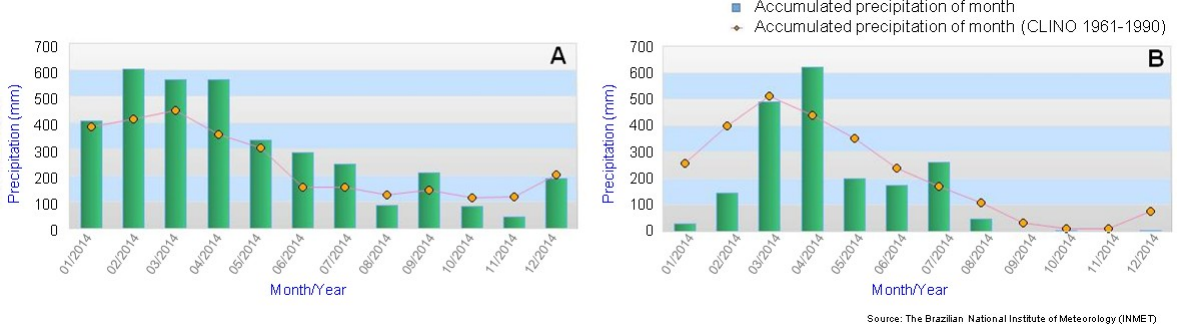
Accumulated precipitation of month X precipitation (CLINO 1961–1990) of the Belém (A) and Tracauateua (B) PCDs.

When comparing the climates of the three areas it was observed that the predominant climate is megathermal and humid with average annual temperature around 27°C. The months of October, November and December are the hottest, with temperatures between 32°C and 34°C and absolute maximums around 41°C. Annual rainfall is quite high, generally around 2,350 mm, but strongly concentrated from January to June (80%). From September to December, on the contrary, rainfall is rare, about 7%, with a short dry season, of moderate water deficit in those months. The relative humidity of the average air oscillates around 85%, as shown in Fig 6 [48].

The description of the accumulated precipitation in the year of capture of the fecal specimens compared to the Climatological Normals (CLINO) for the period from 1961–1990 of the PCDs closest to the locations of the Expedito Ribeiro / Santa Bárbara settlement (Belém PCD), Vila Ananim / Peixe-Boi and Açaiteua / Viseu (Tracauateua PCD) show the frequency of rainfall in the regions, which facilitates the renewal of the pastures and the regeneration of the impacted forests, being an important indicator of the reduction of the damages caused by deforestation in the region.

**Fig 7:**
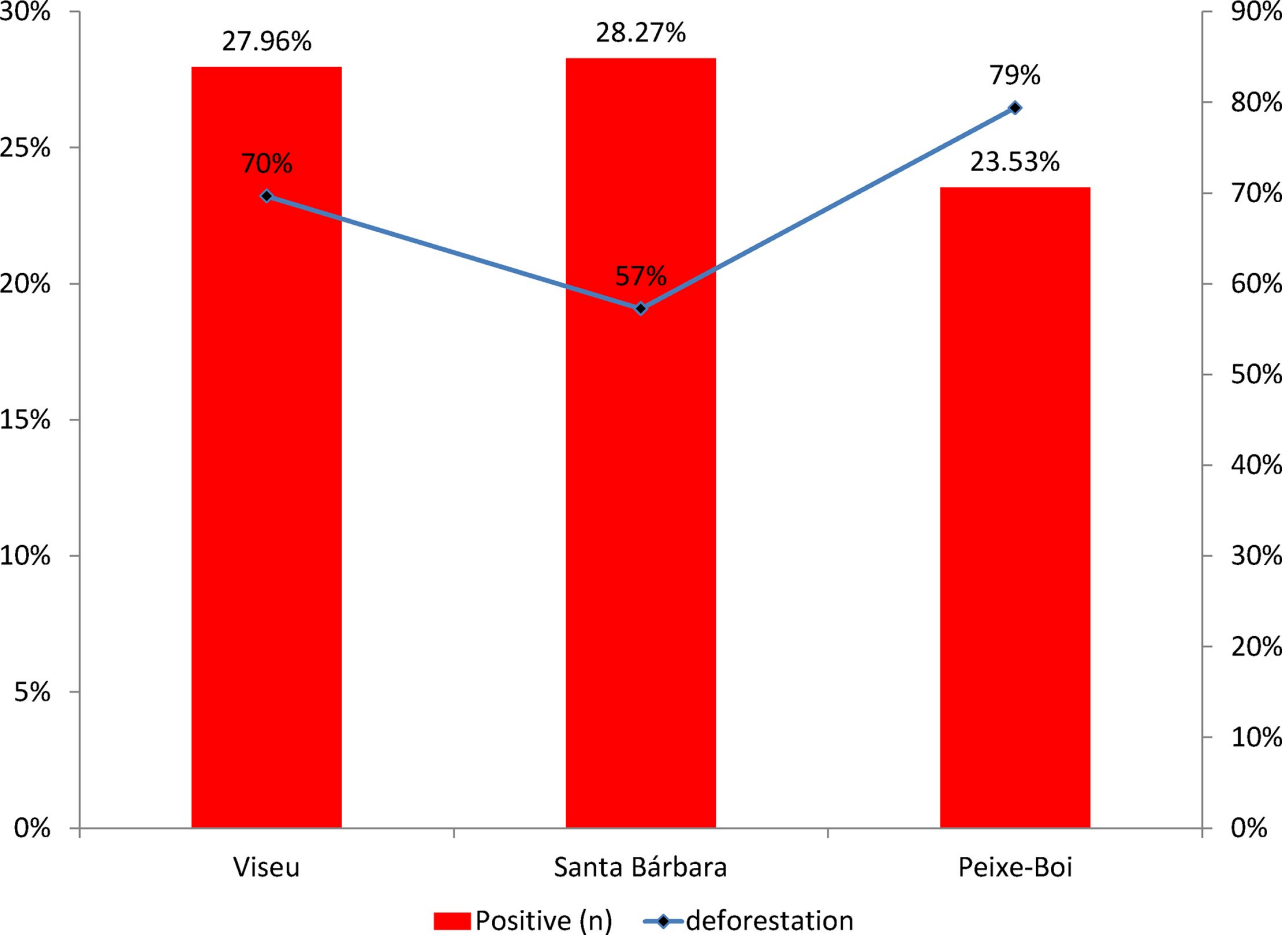
Positivity to RVA correlated with deforestation in the three cities of the Brazilian Amazon. Source: PRODES (http://www.dpi.inpe.br/prodesdigital/prodesmunicipal.php).

The average deforestation index in the three study areas was calculated from data obtained from INPE information systems. It was observed that in the years of 2013 to 2014 there were no changes in these regions; in the period from 2014 to 2015 about 4.1% of the city of Viseu was changed and 1.6% of the city of Peixe Boi. In relation to the period of 2016, great changes were observed in Peixe Boi (79%) and in the city of Viseu (70%), thus demonstrating that changes in the natural ecosystem may be associated with the frequencies for RVA in the studied areas, according to Fig 7.

When assessing the infected animals in relation to the uninfected animals in both the forest fragment and the peridomicile, considering as animals of the forest fragment the birds, the chiroptera and the small rodents and as animals of the peridomicile the canines, bovines, pigs, felines and horses, a percentage of 37.07% infected peri domestic animals (86/232) and 22.12% infected forest fragment animals (92/416) were obtained. Applying the selected statistical analysis, a Pearson x2 Chi-square value was obtained: 16.7159, df = 1 and p <0.001, meaning that the hypothesis was corroborated, that is, the greater the degradation of the environment, the more likely it will be the search for food by wild animals in adjacent areas, or in the edge of the forest or even in the peri domiciliary region. In this sense, the possibility of contagion with other species of animals, even humans, should be considered because of the capacity of the rotavirus to be transmitted via the fecal / oral route or through direct contact with the environment. It is important to point out that the animals detected in this study are important sources of viral strains.

**Fig 8:**
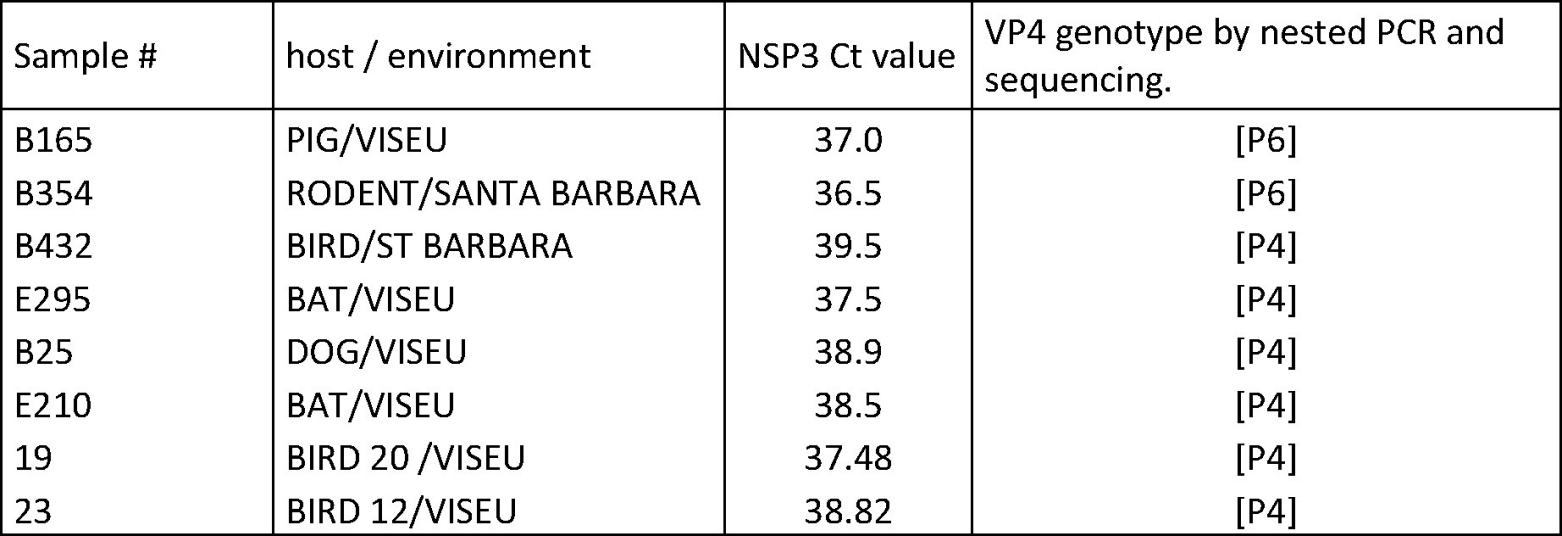
Results of nested and qRT-PCR for samples characterized in accordance to the host and localization.

A total of 80 stool samples were selected, reextracted and analyzed using PCR for the VP4 gene. Eight strains (10%) were positive for VP4 gene, being 2 strains bellowed to P[6] genotype and 6 to P[4]-type, according to Fig 8.

## Discussion

In the present study, RVA was detected circulating in 27.5% of the animals; 36% in domestic animals and 64% in wild animals, providing a unique dataset with qRT-PCR detecting a low viral load of RVA in different species, which further correlates with the deforestation index. These data are important because there is a lack of tests for RVA diagnosis in animals, since the current methods of RVA detection does not always detect in these populations [8]. With the advent of real-time PCR (qPCR), there was an exponential growth, compared to conventional PCR essays, since its superior accuracy, sensitivity and specificity is remarkable, and it is possible to detect RVA in a variety of animal species using NSP3 gene [49]. The sensitivity of RT-qPCR significantly improved the rate of RVA detection in clinical samples from animals and in this context, the present study proposed an interesting study metrics using virus spreading in the wild animals which inhabit forest fragments to indicate human population interventions, with the goal of preventing the virus outbreaks leveraged on the unique geographic characteristics of Brazil and its large number of species in Amazon.

Currently, no data have been described in the literature regarding the RVA detection using real-time qPCR technique in a wide variety of wild animal species. However, a study by Soltan et al. [50] conducted with horses and cattle detected RVA by RT-PCR, commercial RT-PCR and RT-qPCR in 36.7%, 51.4% and 56.9% respectively, differently from the present study that showed higher positivity for chiropterans (17.98%), canines (21.35%), birds (23.6%) and cattle (14.6%).

The first description of RVA in chiroptera was recorded in feces of *Eidolon helvum* caught in Vihiga, Kenya [51]. Afterwards, several strains of RVA were detected by different molecular techniques involving chiroptera, in several countries, including Kenya (*E*. *helvum*), China (*Rhinolophus hipposideros* and *Aselliscus stoliczkanus*), France (*Myotis mystacinus*), Cameroon (*E*. *helvum*) and Brazil [31, 51–55]. The present study shows the occurrence of RVA in 17.98% of the chiroptera, being among the species *Carollia perspicillata* (37.5%), *Desmodus rotundus* (28.12%), *Uroderma bilobata* (15.6%), *Artibeus lituratus* (9.37%), *Artibeus Planirostus* (3.12%), *Diaemus iyoug* and *Glossophagine* (3.12%).

Barquez et al. [56] reported that *Desmodus rotundus* is one of the three hematophagous species of the *Phyllostomidae* family, found throughout South America, Central America and Mexico. Of the positive chiroptera for RVA in the present study, a prevalence of 28.12% was of *Desmodus rotundus*. This species feeds on birds, can feed on mammals, mostly medium or large, facilitating the dissemination of viral spores among the community within the habitat, as observed in the present study. These findings show the importance of epidemiological data on the studied species due to the lack of studies involving species of neotropical chiroptera, and it is not possible to establish comparative parameters for these animals.

Regarding the circulation of RVA in canines and birds, the prevalence was 53% and 29%, respectively. Although in the Amazon region there are records of RVA, RVD, RVF and RVG that infect birds [57–58] and RVD in migratory birds [59], all were detected by RT-PCR assays differently from the present study which detected the RVA by RT-qPCR involving a variety of animal species.

On the other hand, the prevalence in felines (16%) and pigs (22%) was lower, probably because there are few animals of these species in the region, as well as few creations.

The study detected the presence of RVA in different species of animals both in areas near the home and in areas located in fragments of forest, characterized as forest remnants, since they were located in cities that suffered high environmental impacts due to vegetal extractivism, pasture formation for cattle breeding, exploitation of natural resources, and direct reflexes on the habitats of wild animals that can serve as virus sources, thus facilitating the dispersion of RVA among communities of coexisting animals.

It is worth emphasizing that these animals have a greater contact with the human populations of the studied areas since they cohabit with the humans in the region, besides having a high flow of movement between the forest extracts and environments chosen for the present study. However, it is noteworthy that only in the communities of Santa Bárbara and Viseu were collected fecal specimens from asymptomatic humans for diarrhea and tested for RVA, but all were negative. It is notorious yet, the existence of different levels of degradation in the studied environments, considering the presented data. The fragmentation of the forest generates many consequences on the Amazonian biota, being able to alter the diversity and the composition of the animal communities in the fragments and even to interfere in the ecological processes, without considering that the fragments of forest in the Amazon are influenced by the climate, possibly facilitating the dispersion of pathogens by the environment, since the wild animals detected in the present study are asymptomatic and have low viral load for RVA.

The occurrence of RVA in this population of animals may explain the possibility of dispersion of viral strains, since there is a proximity to the human population, besides the biological characteristics of these species that may represent important sources for gastroenteric viruses, along with the fact that all animals were asymptomatic for diarrhea.

Wild birds have unlimited flight capacity, were captured in an interface region between the peridomicile and forest fragments and it is believed that this region has not been influenced by anthropic activities such as those observed in the area of the present study. On the other hand, the breeding method for poultry and canines close to homes and the forest ecosystem, as they are created in the communities surveyed, probably facilitates direct contact with possible sources of contamination, since in the areas the use of septic tanks is deficient and sometimes non-existent, which may facilitate or even increase the risk of viral dispersion throughout the environment.

The high rates of increase and the analysis of land use in the researched areas may be important indicators of how these animals interact, since with deforestation, the populations of wild animals seek refuge in nearby communities facilitating the dispersal of infectious agents and the possible occurrence of carrier animals by direct contact or contamination of the local environment.

To our knowledge, this is the first study in which a real-time PCR assay was applied for the detection of RVA involving a wide variety of domestic and wild animals, facilitating practical utility in epidemiological and molecular studies and assisting in a perspective in the elaboration of sanitary control and monitoring, preventing possible outbreaks in the studied communities. The detection of positive animals was useful to monitor the infection of the agent in the animal population and to provide an early warning signal to predict an impending epidemic and a favorable risk for the human population, given the evidence of RVA circulation in the different forest fragments.

In addition, the RT-qPCR assay may be a useful alternative for the differential diagnosis of RV in possible coexisting mixed infections clinically indistinguishable such as those caused by other viral strains that cause gastroenteritis such as: astrovirus, coronavirus, picobirnavirus, calicivirus, among others as observed in the studies of Jing et al. [60] and Waruhiu et al. [61].

Diarrhea associated with RVA infections in pigs is an important cause of increased mortality and economic losses in Europe. The most prevalent genotypes isolated from feces of Belgian diarrheal and non-diarrheal piglets in 2012 [62] demonstrate a wide range of combinations of genotypes G / P including; G3P[6], G4P[6], G5P[6], G4P[7], G5P[7], G9P[7], G9P[13] and G9P[23]. On the other hand, in the present study it was possible to detect only P[6] genotype, since majority of samples was asymptomatic for diarrhea.

Finding shows that different P genotypes of RVA strains interact with distinct blood group histological antigens (HBGA, ABOH, Lewis) and sialic acids via VP4 providing insight into the regional prevalence and increased zoonotic potential of some RVA of origin swine [63]. The genotype P[6] was identified in piglets in Brazil [64] and in Italy and Japan resembling genotype P[6] human [65, 66].

In the population of animals studied the zoonotic transmission can be frequent, since the animals live in contact with humans and in precarious sanitary conditions. In Brazil, this genotype was described in animal and human populations in studies of Luchs et al. [32]; Honma et al. [67]; Araújo et al.[68]; Mascarenhas et al.[69] and Lorenzetti et al. [70] such studies corroborate the importance of continuing to monitor genotypes to verify if uncommon strains or new strains are emerging and can infect animal populations or inter-species transmissions.

Regarding the genotype P [4], itwas most detected in our samples in bats, dogs, swine and feline. This genotype is not common in animals, being more detected in human and environmental samples in various parts of the world and included our region [71]. It is important to emphasize that the indicators of environmental contamination in Brazil are significant and contribute to the possibility of human-animal transmission [71]. Such data need further investigation in later work to better characterize the interspecies transmission, since the occurrence of enteric viruses in different matrices demonstrates the anthropogenic impact of the exposed population around and points to the potential risk of infection by the possible exposure of individuals susceptible. Our findings may be useful for tracking fecal contamination in the environment using animals as possible sources thus minimizing the risk of infection by exposure to susceptible individuals, in this case different animal species or even human populations.

## Conclusions and Significance

RVA were detected in wild and domestic animals using a RT-qPCR assay that analyzed samples that had low viral load for RVA. Although the samples are asymptomatic for diarrhea, it is necessary to conduct strategies for the monitoring and control of the animals in the areas studied in the human population as well as in other species of animals, as well as the implementation of preventive measures aimed at future outbreaks in communities animals in the resident population in these impacted areas. Therefore, the present study is unprecedented in the region and in the country in relation to the research of RVA in wild animals. It is noteworthy that, although the quality of the analyzed samples is characterized as low detectable viral load, the technique presented a good analytical response in the detection of the source animals for RVA, facilitating the selection of the samples for future genetic characterization tests.
