# Correction: Rotavirus A in wild and domestic animals from areas with environmental degradation in the Brazilian Amazon

**DOI:** 10.1371/journal.pone.0211311

**Published:** 2019-01-23

**Authors:** Bruno de Cássio Veloso de Barros, Elaine Nunes Chagas, Luna Wanessa Bezerra, Laila Graziela Ribeiro, Jose Wandilson Barboza Duarte Júnior, Diego Pereira, Edvaldo Tavares da Penha Junior, Julia Rezende Silva, Delana Andreza Melo Bezerra, Renato Silva Bandeira, Helder Henrique Costa Pinheiro, Sylvia de Fátima dos Santos Guerra, Ricardo José de Paula Souza e Guimarães, Joana D’Arc Pereira Mascarenhas

Fig 2 is incorrect. The language of Fig 2 is incorrectly in Portuguese. The authors have provided a corrected version in English here.

**Fig 2:**
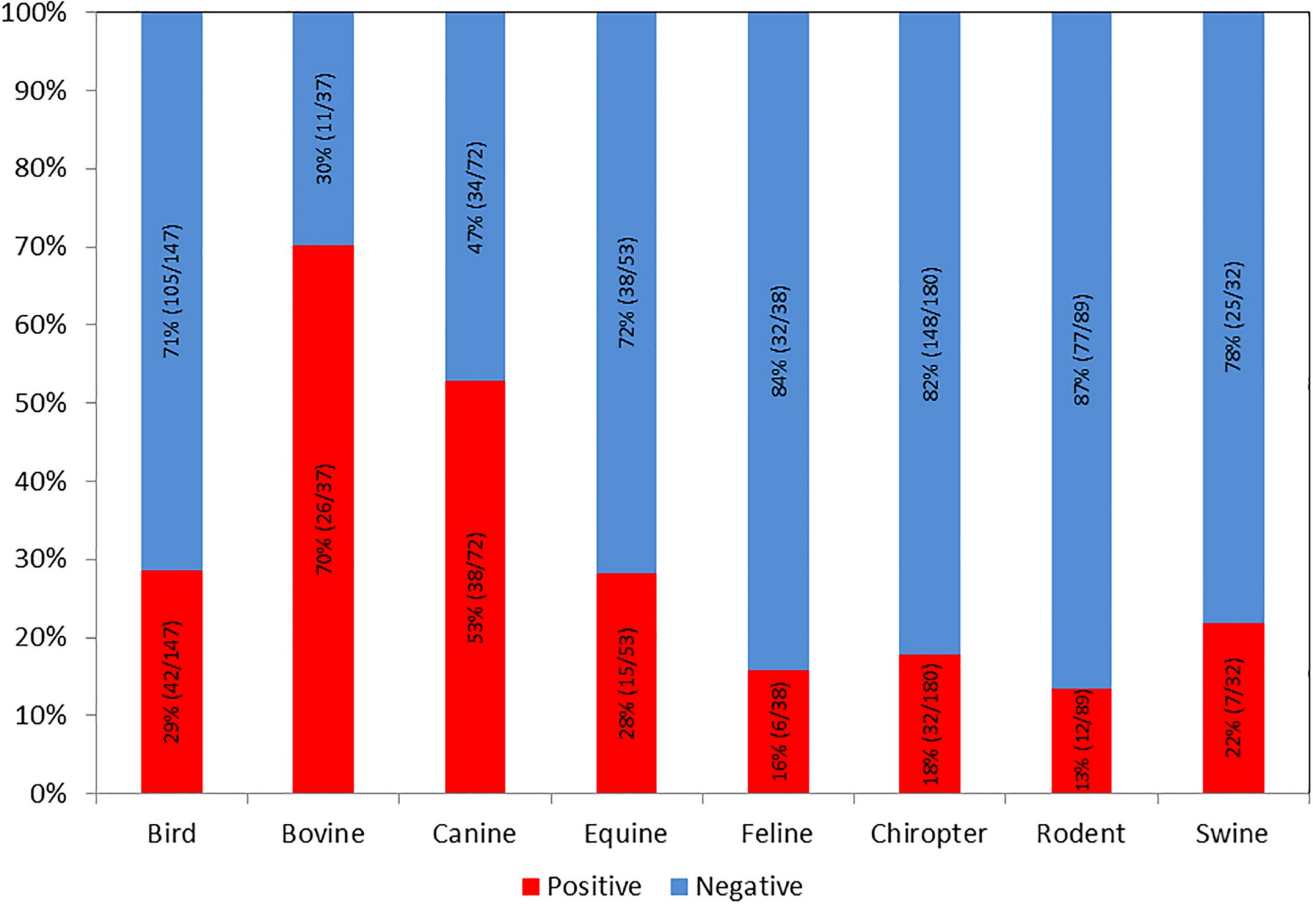
Frequency of Rotavirus A in domestic and wild animals in the Brazilian Amazon from 2014 to 2016.
